# Combination with a Low Dose of Doxorubicin Further Boosts the Antitumor Effect of SLURP-1 In Vivo and Associates with EGFR Down-Regulation

**DOI:** 10.32607/actanaturae.27526

**Published:** 2025

**Authors:** O. V. Shlepova, M. L. Bychkov, V. O. Shipunova, E. I. Shramova, M. A. Shulepko, T. Y. Gornostaeva, E. A. Kiseleva, I. D. Kukushkin, V. A. Kazakov, E. A. Tukhovskaya, I. A. Dyachenko, A. N. Murashev, Z. O. Shenkarev, S. M. Deyev, M. P. Kirpichnikov, E. N. Lyukmanova

**Affiliations:** Shemyakin-Ovchinnikov Institute of Bioorganic Chemistry, Russian Academy of Sciences, Moscow, 117997 Russian Federation; Moscow Center for Advanced Studies, Moscow, 123592 Russian Federation; Faculty of Biology, MSU-BIT Shenzhen University, Shenzhen, 518172 China; Branch of Shemyakin-Ovchinnikov Institute of Bioorganic Chemistry RAS, Pushchino, 142290 Russian Federation; Biomarker Research Laboratory, Institute of Fundamental Medicine and Biology, Kazan Federal University, Kazan, 420008 Russian Federation; National Research Ogarev Mordovia State University, Republic of Mordovia, Saransk, 430005 Russian Federation; Interdisciplinary Scientific and Educational School of Moscow University “Molecular Technologies of the Living Systems and Synthetic Biology”, Faculty of Biology, Lomonosov Moscow State University, Moscow, 119234 Russian Federation

**Keywords:** cancer, chemotherapy, SLURP-1, Ly6/uPAR, α7-nAChR, EGFR

## Abstract

Skin cancers such as squamous cell carcinoma (SCC) are among the most aggressive types of tumors. They come with a high rate of growth, metastasis, and frequently occurring chemoresistance. Smoking is one of the risk factors for SCC progression, and the α7 nicotinic acetylcholine receptor (α7-nAChR) is a promising target for SCC therapy. Human secreted protein SLURP-1 is an auto/paracrine regulator of epithelial homeostasis and a selective negative allosteric modulator of α7-nAChR. Recently, we demonstrated the high efficiency of the therapy based on the recombinant SLURP-1 in controlling SCC cell growth and metastasis *in vivo*. The anti-tumor effect of SLURP-1 was mediated through interaction with both α7-nAChR and the epidermal growth factor receptor (EGFR). Cytotoxic antibiotic doxorubicin has been proposed for the SCC therapy; however, its use is limited due to the high toxicity. In this study we investigated the use of an enhanced SLURP-1 dose and of a combination of SLURP-1 with low-dozen doxorubicin for SCC treatment of mice xenografted with squamous cell carcinoma A431 cells. An increased SLURP-1 dose didn’t significantly enhance the efficiency of the therapy. However, the combination with doxorubicin further enhanced the anti- tumor activity of SLURP-1 and dramatically suppressed metastasis. The effect from the combined therapy was accompanied by down-regulation of EGFR expression in tumors. Direct inhibition of EGFR activation by SLURP-1 was shown. No toxicity of the combined therapy was encountered. Our data indicate that the combination of SLURP-1 with chemotherapy in lower doses is a promising approach in SCC treatment and should be further studied.

## INTRODUCTION

Skin cancer, particularly squamous cell carcinoma (SCC), is one of the most aggressive types of tumors, as its incidence, morbidity, and mortality rates continue to increase worldwide [1]. The major obstacles in the treatment of SCC are the inability to achieve a complete surgical removal of the tumor, tumor metastasis, and the development of resistance to chemotherapeutic agents [1, 2, 3, 4]. Smoking is one of the risk factors for SCC progression [5], and nicotinic acetylcholine receptors (nAChRs) activated upon tobacco consumption are promising targets for SCC therapy. nAChR of α7 type (α7-nAChR) is well known as a tumor growth promoter [6, 7, 8, 9]. The expression of α7-nAChR is increased in cancer cells compared to normal cells [10], and it correlates with a poor prognosis [11, 12]. Activation of α7-nAChR promotes the proliferation, angiogenesis, migration, and invasion of carcinoma and glioma cells [8, 12, 13, 14, 15, 16, 17, 18, 19]. In cancer cells, α7-nAChR can form heteromeric complexes with another prooncogenic receptor: the epidermal growth factor receptor (EGFR) [20, 21, 22, 23]. Moreover, activation of α7-nAChR in SCC by nicotine promotes chemoresistance and metastasis via the transactivation of EGFR [24].

Some endogenous human proteins of the Ly6/uPAR family [25] modulate the α7-nAChR activity and can be considered prototypes for tumor-selective and nontoxic targeted anticancer drugs. The human secreted protein SLURP-1 is one of such α7-nAChR modulators [26] and an auto/paracrine regulator of epithelial homeostasis [27]. SLURP-1 expression is down-regulated in primary and metastatic melanoma compared to normal cells [28, 29], while an elevated plasma level of SLURP-1 correlates with a better chance of survival for patients with pancreatic cancer [30]. A recombinant analogue of SLURP-1 inhibits cancer cell growth *in vitro *and *in vivo *[21, 22, 30, 31, 32, 33, 34, 35], as well as abolishes nicotine-induced cell proliferation [36]. Its anti-tumor effect *in vivo *in the SCC model (A431 xenografts) is mediated by an interaction with both α7-nAChR and EGFR [22].

Doxorubicin (a DNA-intercalating anthracycline antibiotic that also inhibits EGFR signaling [37, 38]) has been proposed for SCC therapy [39], because it appears to exert a complex, antiproliferative effect by inhibiting the transcription of oncogenes and generating free radicals [40]. However, its use in therapy is severely limited by its high toxicity [41]. Thus, a reduced dose of doxorubicin can be a good way to counteract its possible side effects.

Here, we propose using lowered concentrations of doxorubicin in combination with SLURP-1. We investigated whether a combination of low-dose SLURP-1 and doxorubicin could be used to control the growth and metastasis of SCC cells *in vivo*. Beside the high efficiency of the proposed therapy, a decreased EGFR expression in tumors of mice treated with SLURP-1 and doxorubicin was revealed. The data obtained indicate the high potential of the proposed approach.

## EXPERIMENTAL

**Materials and animals**

Recombinant SLURP-1 was produced in *E. coli *as previously described [31, 42].

Doxorubicin was provided by TEVA (Tel Aviv-Yafo, Israel).

The animals were bred and housed under the standard conditions of the Animal Breeding Facility, BIBCh, RAS, accredited at the international level by AAALACi. All procedures were performed in accordance with the ethical recommendations of Rus- LASA approved by the Institutional Animal Care and Use Committee of IBCh, RAS (protocol # 318/2021).

**Cell cultivation and migration analysis by scratch assay**

Human squamous cell carcinoma A431 cells (ATCC, Manassas, VA, USA) were grown (37°C, 5% CO_2_) in a DME medium (PanEco, Russia), 10% fetal calf serum (Thermo Fisher Scientific, USA), abbreviated as the complete medium. The cells were subcultured at least twice per week.

Cell migration was measured by a scratch assay as described earlier [21, 43]. Images were obtained using CloneSelect Imager (Molecular Devices, United States), and the scratch area occupied by migrating cells was quantified using ImageJ (NIH, United States). Data were normalized to the average area occupied by migrated cells in the control wells and approximated with a Hill equation.

**Tumor xenograft model, treatment strategy, and living mice imaging**

To obtain the luminescent A431/NanoLuc cells, the parental A431 cells were transfected with the NanoLuc plasmid as described in [44] using the FuGENE HD transfection reagent (Promega, USA).

**Fig. 1:**
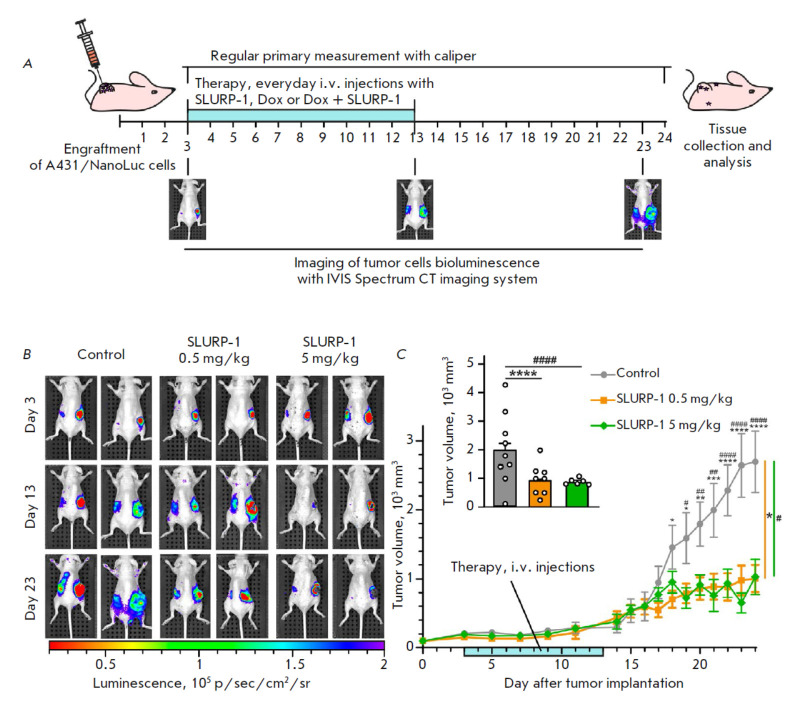
The influence of different SLURP-1 doses on tumor growth in the A431/NanoLuc mice xenograft model. (*A*) Scheme of drugs administration and tumor growth measurements. (*B*) Representative images of tumor bioluminescence (A431/NanoLuc cells) before treatment (the 3^rd^ day after tumor engraftment, the 1^st^ day of the therapy), after treatment (the 13th day after tumor engraftment, the next day after end of the 10-day therapy course), and before sacrification (the 23^rd^ day after tumor engraftment). See *Fig. S1 *for all mice images. (*C*) The primary tumor volume measurements with a caliper. Data presented as mm3 ± SEM. *(*p* < 0.05), **(*p* < 0.01), ***(*p* < 0.001), and ****(*p* < 0.0001) indicate a significant difference between the Control (saline) and (0.5 mg/kg SLURP-1) groups; #(*p* < 0.05), ##(*p* < 0.01), and ####(*p* < 0.0001) indicate a significant difference between the Control and (5 mg/kg SLURP-1) groups according to the two-way ANOVA, followed by Dunnett’s post hoc test. The days of treatment are marked with a light blue bar; (*C*, insert). The average primary tumor volume measured with a caliper for each mouse in the last 5 days (20–24 days after tumor engraftment). Data are presented as mm3 ± SEM. ****(*p* < 0.0001) and #### (*p* < 0.0001) indicate the significant difference between the Control and groups according to one-way ANOVA followed by Tukey‘s post hoc test

Male BALB/c Nu/Nu mice (22–25 g) were engrafted subcutaneously on the back with 107 A431/NanoLuc cells in 100 μL of 30% Matrigel (Corning, USA) in the complete medium. On the 3^rd^ day after A431/NanoLuc cells engraftment, the mice were randomly divided into five groups (initially n = 8–10, *Table S1*), and i.v. injected once a day for the ten subsequent days with 100 µL of a 0.9% NaCl solution (saline) containing: 1) no additives –control, 2) 100 µg of SLURP-1 (final body concentration 5 mg/kg), 3) 10 µg of SLURP-1 (final body concentration 0.5 mg/kg), 4) 50 µg of doxorubicin (2.5 mg/kg), 5) 5 µg of doxorubicin (final body concentration 0.25 mg/kg) with 10 µg of SLURP-1 (final body concentration 0.5 mg/kg) (*Fig. 1A*). Some animals died during the experiment (*Table S1* and *Fig. S1*) and were excluded from the analysis.

The primary tumor volume was measured with a caliper and calculated using the formula

*V *= 0.52 × *A *× *B*^2^

(*A *is the largest diameter and *B *is the smallest diameter).

On the 3^rd^, 13th, and 23^rd^ days after tumor engraftment, tumors were visualized with the IVIS Spectrum CT imaging system (Perkin Elmer, USA) as described earlier [22]. Bioluminescence images were acquired using a IS1803N7357 iKon camera (Andor, Belfast, UK) and normalized to photons per second per cm^2^ per steradian (p/sec/cm2/sr) and analyzed using the Living Image 4.5.5.19626 software (Xenogen, USA).

On the 24^th^ day after tumor engraftment, the mice were euthanized by cervical dislocation, and the tumors were isolated with a scalpel and forceps and immediately frozen at –150°C for further analysis. The lungs, liver, kidneys, spleen, and heart were removed from the euthanized mice with a scalpel and forceps and placed in a 4% paraformaldehyde solution (Applichem, Spain).

**Western blotting**

To assess the influence of SLURP-1 and doxorubicin on EGFR expression, the tumors (0.05 mg per sample) were homogenized, solubilized in 2% Triton X-100, and diluted in non-reducing PAGE buffer. Western blotting was performed with primary antibodies (sc-120, Santa Cruz, USA, 1 : 1 000) and secondary antibodies (715-035-150, Jackson Immunoresearch, USA, 1 : 5 000) for EGFR detection. The HRP signal was detected with the ECL substrate (Bio-Rad, USA) using an ImageQuant LAS 500 chemidocumenter (GE Healthcare, USA). Data were processed using the ImageJ 1.53t software (NIH, USA).

**In-cell ELISA**

To study the effect of SLURP-1 on EGFR activation, A431 cells were seeded in 96-well culture plates (1 × 10^4^ cells/well). After 24 h the culture medium was replaced with a serum-free medium, and after another 24 h the culture medium was changed to ones containing SLURP-1 at various concentrations. Preincubation with SLURP-1 was performed for 30 min. After that, EGFR activation was stimulated by the addition of 25 nM EGF to the cells, which were incubated for another 3 h at 37°C, 5% CO_2_. The cells were fixed with a 4% paraformaldehyde solution in PBS, blocked with PBS buffer containing 2% BSA and 0.1% Triton X-100, and incubated with primary antibodies against p‑EGFR(Y1173) (ABIN343717, antibodies- online, 1 : 1 000) and with secondary antibodies (715-035-150, Jackson Immunoresearch, West Grove, PA, USA, 1 : 5 000). Next, 50 μL of a TMB solution was added to the wells. The reaction was stopped with a 2M H_2_SO_4_ solution, and the absorbance in the wells was determined at 450 nm using a AMR-100 plate reader (Allsheng, China).

**Histochemistry**

For the histochemical analysis, samples of the lung, liver, kidney, spleen, and heart from three randomly selected mice from each group that had received saline (control), SLURP-1 (5 mg/kg), doxorubicin (2.5 mg/kg), or SLURP-1 (0.5 mg/kg) + doxorubicin (0.25 mg/kg) were fixed in a 10% neutral formaldehyde solution in PBS buffer, washed in running tap water, dehydrated in graded alcohols, and embedded in paraffin. The 4- to 5-μm-thick Paraffin sections stained with hematoxylin and eosin were examined with a conventional light AxioScope.A1 microscope (Carl Zeiss, Germany). Microphotographs of the histologic preparations were taken with the high-resolution camera Axiocam 305 color (Carl Zeiss) equipped with the ZEN 2.6 lite software (Carl Zeiss) at ×200 magnification.

**Statistical Analysis**

Data are presented as a mean ± SEM. The number of samples (n) is indicated in the figure legends. The statistical analysis was performed using the GraphPad Prism 9.5.0 software (Graphpad software, USA). The data were analyzed for a normal distribution using the Shapiro-Wilk omnibus normality test. For nonparametric data, the Kruskal-Wallis test was used, instead of the one-way ANOVA test. The analysis was performed using the unpaired t-test; the Kruskal- Wallis test, followed by Dunn’s post hoc test; one-way ANOVA, followed by Dunnett’s or Tukey’s post hoc test; one-way Welch ANOVA, followed by Dunnet’s post hoc test; and two-way ANOVA, followed by Dunnett’s post hoc test as indicated in the figure legends. Differences between groups were considered statistically significant at *p* < 0.05.

## RESULTS

**An increased dose of SLURP-1 doesn’t increase the therapeutic efficiency *in vivo***

In this work we compared two doses of the protein: we used the 0.5 mg/kg used in [22] and the ten times higher 5 mg/kg dose in the same xenograft mouse model of human epidermoid carcinoma used as described previously [22]. Surprisingly, the effect of the higher dose of SLURP-1 did not differ from that achieved with the lower dose (*Fig. 1B,C*). The 0.5 mg/kg and 5 mg/kg doses of SLURP-1 both inhibited primary tumor growth (*Fig. 1A-C*, *S1*) with similar efficacy, with a ~ 3-fold reduction in the primary tumor volume compared to the control (*Fig. 1C*, insert). Thus, the experiment demonstrated that the effect of SLURP-1 hit a ceiling and could not be enhanced by increasing the dose.

**Low doses of the SLURP-1 /doxorubicin combination have an additive antimigratory effect *in vitro***

**Fig. 2:**
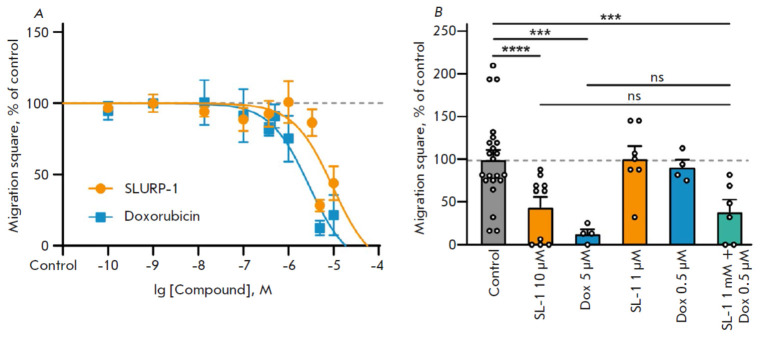
The influence of different SLURP-1 and doxorubicin doses on A431 cells migration. (*A*) Effect of different SLURP-1 and doxorubicin concentrations on cell migration. Data are presented as the mean scratch surface occupied by migrating cells (% normalized to the control), ± SEM, *n *= 3–22. The data obtained was approximated using a Hill equation. The Control level (100%) is shown with a dashed line. (*B*) Effect of SLURP-1 (SL-1) and doxorubicin (Dox) and their combination on cell migration. Data are presented as the mean scratch surface occupied by migrating cells (% normalized to control), ± SEM, *n *= 3–22; Control level (100%) is shown by dashed line. ***(*p* < 0.001), and ****(*p* < 0.0001) indicate a significant difference from the control group (untreated cells) by one-way ANOVA followed by Dunnett’s post hoc test, “ns” means no significant difference between the groups

Previously, using multicellular spheroids reconstituted from A549 and A431 cells, we observed the additive antiproliferative effect of doxorubicin (a widely used cancer chemotherapy drug [45]) and SLURP-1* in vitro *[46]. Here, we observed a strong dose-dependent reduction of cell migration after 24 h incubation with SLURP-1 or doxorubicin with EC_50_ 9.4 ± 7.8 µM and 2.3 ± 1.7 µM, respectively (*Fig. 2A,B*, *Table S2*). Notably, 10 µM of SLURP-1 is equivalent to the 5 mg/kg dose used *in vivo*, and 5 µM of doxorubicin is equivalent to 2.5 mg/kg (equivalent to the 25 mg/kg cumulative dose (75 mg/m2) recommended for one cycle of solid tumor therapy (60 mg/m2) [47]). The combination of 1 µM SLURP-1 and 0.5 µM doxorubicin resulted in robust cell migration inhibition compared to the effects of 10 µM of SLURP-1 or 5 µM doxorubicin taken alone (*Fig. 1B*). Thus, the combination of low doses of SLURP-1 and doxorubicin has an additive effect on A431 cell migration.

**Combination with low-dose doxorubicin increases the antitumor activity of SLURP-1 *in vivo***

**Fig. 3:**
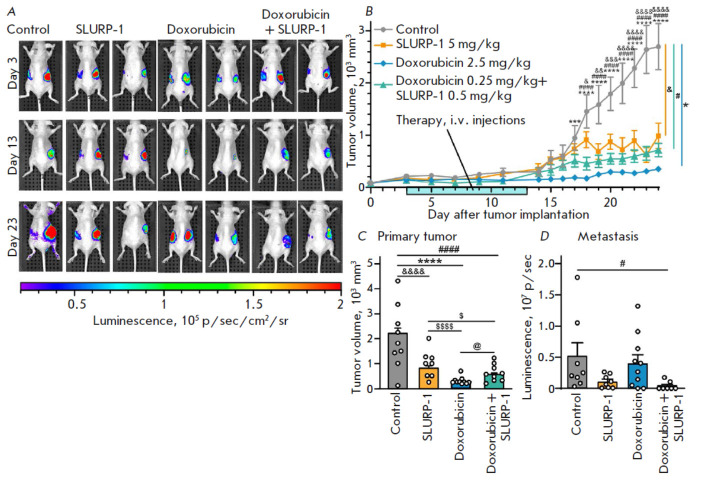
The influence of SLURP-1, doxorubicin, and their combination on tumor growth and metastasis in a A431/NanoLuc mice xenograft model. (*A*) Representative images of tumor bioluminescence (A431/NanoLuc cells) before treatment (the 3^rd^ day after tumor engraftment, the 1^st^ day of therapy), after treatment (the 13th day after tumor engraftment, the next day after conclusion of the 10-day therapy), and before sacrification (the 23^rd^ day after tumor engraftment). See *Fig. S1 *for all mice images. (*B*) The primary tumor volume measurements with a caliper. Data are presented as mm3 ± SEM. **(*p* < 0.01) and ****(*p* < 0.0001) indicate a significant difference between the Control (saline) and (2.5 mg/kg doxorubicin) groups; #### (*p* < 0.0001) indicates a significant difference between the Control and (0.5 mg/kg SLURP-1 + 0.25 mg/kg doxorubicin) groups; &(*p* < 0.05), &&(*p* < 0.01), &&&(*p* < 0.001), and &&&&(*p* < 0.0001) indicate a significant difference between the Control and (5 mg/kg SLURP-1) groups according to two-way ANOVA followed by Dunnett’s post hoc test. The days of treatment are marked with a light blue bar. (*C*) The average primary tumor volume measured with a caliper for each mouse for the last 5 days (20–24 days after tumor engraftment). Data presented as mm3 ± SEM. ****(*p* < 0.0001) indicates a significant difference between the Control and (2.5 mg/kg doxorubicin) groups; #### (*p* < 0.0001) indicates a significant difference between the Control and (0.5 mg/kg SLURP-1 + 0.25 mg/kg doxorubicin) groups; &&&&(*p* < 0.001) indicates a significant difference between the Control and (5 mg/kg SLURP-1) groups; $(*p* < 0.05) indicates a significant difference from the (5 mg/kg SLURP-1) group; and @(*p* < 0.05) indicates a significant difference between the (2.5 mg/kg doxorubicin) and (0.5 mg/kg SLURP-1 + 0.25 mg/kg doxorubicin) groups according to one-way ANOVA followed by Tukey’s post hoc test. (*D*) Total luminescence measured in the areas outside of the primary tumor. Data are presented as photons per second (p/sec) ± SEM. #(*p* < 0.05) indicates a significant difference from the Control (saline) group according to the Kruskal-Wallis followed by Dunn’s post hoc test

Next, we showed that the combination of 0.5 mg/kg SLURP-1 (1 µM *in vitro*) with 0.25 mg/kg of doxorubicin (0.5 µM *in vitro*) reduced primary tumor growth more efficiently than the application of a high dose of SLURP-1 taken alone (*Fig. 3A,B,C*). Moreover, combined usage of SLURP-1 with low-dose doxorubicin significantly suppressed metastasis, while treatment with SLURP-1 (5 mg/kg) or doxorubicin (2.5 mg/kg) alone failed to have any impact on metastasis (*Fig. 3A,B,D* and *Fig. S1*). Thus, it’s reasonable to conclude that SLURP-1 is a perspective anticancer agent for combination therapy in which the dose of the toxic chemotherapeutic agent can be reduced.

**The combination of SLURP-1 with doxorubicin suppresses EGFR expression in tumors *in vivo***

**Fig. 4:**
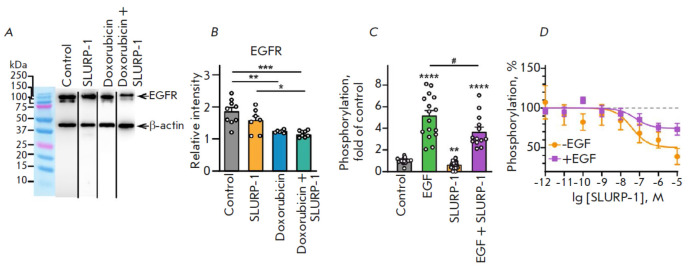
The effect of SLURP-1 on EGFR expression and activation. (*A*) Representative Western blot membrane analysis of the EGFR expression in tumors after treatment with saline (control), SLURP-1 (5 mg/kg), doxorubicin (2.5 mg/kg), or SLURP-1 (0.5 mg/kg) + doxorubicin (0.25 mg/kg). Whole membranes are shown in Fig. S2. The samples shown were run on different membranes in parallel. (*B*) The expression level of EGFR was normalized to the β-actin expression level. Data are presented as the relative intensity ± SEM (*n *= 6–9). **(*p* < 0.01), ***(*p* < 0.001), and ****(*p* < 0.0001) indicate significant differences between the groups per one-way ANOVA followed by Tukey’s post hoc test. (*C*) The effect of 1 μM SLURP-1, 25 nM EGF alone, and their mix on EGFR activation by autophosphorylation at Y1173 in A431 cells. Data are presented as fold of control (untreated cells) ± SEM (*n *= 13–17). **(*p* < 0.01) and ****(*p* < 0.0001) indicate significant differences from Control according to one-way Welch ANOVA followed by Dunnet’s post hoc test. # (*p* < 0.05) indicates significant differences between the groups per the unpaired t-test. (*D*) The effect of different concentrations of SLURP-1 on EGFR activation in the absence and presence of EGF (*n *= 10–14). Data are presented as % of the Control ± SEM. The data obtained was approximated using a Hill equation

EGFR, the best known pro-oncogenic receptor [23], is overexpressed in squamous cell carcinoma A431 cells [48]. In this work, we showed that therapy with either doxorubicin alone (2.5 mg/kg) or in combination with SLURP-1 and doxorubicin (0.25 mg/kg doxorubicin + 0.5 mg/kg SLURP-1) results in a significant decrease in the EGFR expression in xenografted A431 tumors (*Fig. 4A,B*).

**SLURP-1 affects the activation of EGFR**

SLURP-1 dampened the Y1173 autophosphorylation of EGFR expressed in A431 cells. Moreover, a decreased EGF-induced phosphorylation of EGFR was observed in the presence of SLURP-1 (*Fig. 4C,D*, *Table S3*). These effects demonstrated a dose-concentration dependence with similar EC_50_ ~ 40 ± 11 nM and 60 ± 17 nM, respectively, with a significant difference in the maximum effect (50 ± 9% and 74 ± 5%, respectively). The same efficiency in the inhibition of EGFR activation with a changed amplitude of the effect (*Fig. 4D*, *Table S3*) points to the rather different binding sites of EGF and SLURP-1 on the surface of the EGFR molecule.

**Combined SLURP-1 and doxorubicin administration showed no toxicity *in vivo***

**Fig. 5:**
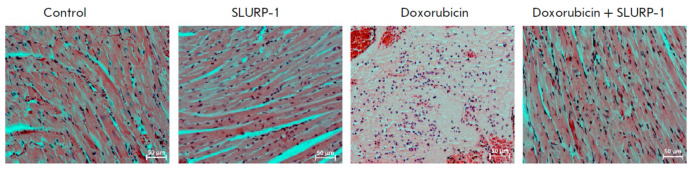
Cardiotoxicity of the SLURP-1 and doxorubicin treatment. Heart fragments of mice treated with saline (control), SLURP-1 (5 mg/kg), doxorubicin (2.5 mg/kg), and SLURP-1 (0.5 mg/kg) + doxorubicin (0.25 mg/kg). Extensive focus of cardiomyocyte necrosis with neutrophil infiltration in the heart of mouse from the doxorubicin group was revealed. Hematoxylin and eosin staining, magnification ×200

To study the potential toxicity of the investigated drugs, organs from mice (three randomly selected mice from each group) were harvested and tests were run for pathological changes. No lung, liver, spleen, kidney, or liver of any animals from any of the groups showed any significant abnormalities that could be attributed to toxicity (*Fig. S3*). At the same time, foci of cardiomyocyte necrosis were found in the hearts of two animals that had received 2.5 mg/kg doxorubicin (*Fig. 5*). Thus, we could conclude that combined therapy with low doses of SLURP-1 and doxorubicin is safer than the use of high doses of doxorubicin alone.

## DISCUSSION

Despite its severe adverse effects, chemotherapy remains the main choice for cancer treatment [49]. One of the most popular chemotherapeutic agents is doxorubicin, which exhibits high antitumor efficacy but also is highly toxic [40, 50]. The toxicity increases with cumulative doses and patient age, which limits the scope of use of the drug [41, 50, 51, 52, 53, 54]. Several studies have proposed therapies featuring a combination of chemotherapy with other approaches to lower the dose of chemotherapy and ease its side effects [55, 56]. Inhibition of α7-nAChR can be considered a promising approach on the road to combined cancer therapy, as it can help reduce tumor progression, metastasis, chemoresistance, and the side effects of chemotherapy [19, 25, 57, 58, 59, 60, 61]. The human secreted protein SLURP-1 negatively modulates the α7-nAChR function [26] and exhibits antitumor activity* in vivo *[22]. Here, we proposed two approaches to improve the efficacy of SLURP-1-based therapy: (1) increasing the dose of SLURP-1 as a monotherapy and (2) a combination of SLURP-1 with doxorubicin.

In keeping with our previous data, SLURP-1 alone was shown to inhibit tumor growth *in vivo*, while a 10-fold increase in the SLURP-1 dose failed to improve the outcome (*Fig. 1*). By testing the second approach, it was shown that low concentrations of SLURP-1 and doxorubicin have an additive antimigratory effect *in vitro* (*Fig. 2B*), as well as anti-tumor and anti-metastastatic effects *in vivo* (*Fig. 3E,F*). Previously, it had been shown through immunogenicity and toxicity tests that SLURP-1 upon intravenous treatment was highly safe [22]. In contrast to SLURP-1, doxorubicin demonstrated elevated cardiotoxicity in mice (*Fig. 5*) at the concentration usually used in clinics [47]. At the same time, a 10-time decrease in the doxorubicin concentration, in combination with SLURP-1, showed no cardiotoxic effects (*Fig. 5*). Thus, the use of low doses of doxorubicin, in combination with SLURP-1 or other inhibitors of α7-nAChR, could be a positive development in antitumor therapy.

The exact molecular mechanisms underlying the combined effect of SLURP-1 and doxorubicin on A431 tumor growth remain unknown. One of the explanations can be a joint inactivation of the EGFR overexpressed in A431 cells [62] by both agents. Indeed, doxorubicin alone, and in combination with SLURP-1, suppresses the expression of this receptor in tumors (*Fig. 4A,B*). EGFR mediates the growth, migration and survival of cancer cells [63]. SLURP-1 cancels the EGF-induced activation of the receptor (*Fig. 4C,D*) by interacting with the α7-nAChR/EGFR complex in A549 and A431 cells [21, 22], and doxorubicin likewise affects the EGFR signaling pathways [38]. On the other hand, the observed orchestra-like interaction between SLURP-1 and doxorubicin can be a result of the inhibition of the complementary intracellular signaling mechanisms. Indeed, overexpression of Src [64], activation of the STAT3 [65] and PI3K/ AKT [66] pathways all lead to the stimulation of EGFR activity and expression in cancer cells. In line, incubation with SLURP-1 leads to inhibition of these signaling pathways in A431 cells [22]. On the other hand, the anti-tumor effect of doxorubicin is mediated by the reorganization of lipid rafts via the EGFR/ Src signaling [38]. Thus, the enhanced combined effect of SLURP-1 and doxorubicin could be a result of synergy between the effects of each compound on the signaling pathways regulating the EGFR expression and activation.

## CONCLUSION

Combination with low-dose doxorubicin enhances the SLURP-1 anti-tumor activity and dramatically suppresses tumor metastasis. The enhanced effect could be associated with down-regulation of EGFR in tumors at the expression and activation levels by both drugs. Thus, combined therapy of tumors, in particularly SCC, by SLURP-1 with low doses of chemotherapeutic agents looks promising and requires further study.

## Abbreviations

AKT: protein kinase B
EGF: epidermal growth factor
EGFR: epidermal growth factor receptor
nAChR: nicotinic acetylcholine receptors
PI3K: phosphoinositide 3-kinase
SCC: squamous cell carcinoma
Src: non-receptor tyrosine kinase Src
STAT3: signal transducer and activator of transcription 3
